# Herbal medicine use by pregnant women in Bangladesh: a cross-sectional study

**DOI:** 10.1186/s12906-018-2399-y

**Published:** 2018-12-13

**Authors:** Mansoor Ahmed, Jung Hye Hwang, Mohammad Ashraful Hasan, Dongwoon Han

**Affiliations:** 1 Department of Biosciences, Shaheed Zulfikar Ali Bhutto Institute of Science and Technology, Karachi, Pakistan; 2 Institute of Health Services Management, Hanyang University, Seoul, South Korea; 3 Department of Global Health and Development & Department of Preventive Medicine, Hanyang University, College of Medicine, 222 Wangsimni-ro, Seongdong-gu, Seoul, 04763 South Korea; 4 Department of Obstetrics and Gynecology, Hanyang University, College of Medicine, Seoul, South Korea; 5 Dhaka Mohanagar General Hospital, South City Corporation, Dhaka, Bangladesh

**Keywords:** Herbal medicines, Pregnancy outcome, Safety, Bangladesh

## Abstract

**Background:**

Herbal medicines in pregnancy are increasingly used worldwide with prevalence of up to 67%. Although this popularity is mainly because of the common belief that these medicines are safe, recent reports suggest that several herbal medicines are potentially harmful to mother and fetus if used in pregnancy.

**Methods:**

This *cross-sectional* study was conducted in July and August of year 2017, at maternity wards of two public hospitals in Dhaka, Bangladesh. Postpartum women were interviewed via the structured questionnaire to collect information regarding socio-demographic and health characteristics, patterns of herbal medicines used in the previous pregnancy, and outcome of pregnancy.

**Results:**

Two hundred forty-three postpartum women participated in the study, with 70% of them using at least one modality of herbal medicines in previous pregnancy. Ginger, black seed, lemon tea, prune, and mustard oil were most commonly used herbal medicines. Herbal medicines were mostly used for pregnancy-related symptoms such as nausea, vomiting, and cold. Fifteen (8.8%) herbal medicine users reported side effects.

**Conclusions:**

This study highlights popularity of herbal medicines during pregnancy in Bangladesh. Previous herbal medicine users and unemployed women turned significantly more to herbal medicines during pregnancy. Reports of side effects and use of some potentially harmful modalities warrant awareness regarding proper use of herbal medicines in pregnancy and its pharmacovigilance.

**Electronic supplementary material:**

The online version of this article (10.1186/s12906-018-2399-y) contains supplementary material, which is available to authorized users.

## Background

Globally, sales of herbs have sharply been increasing, and herbal medicines (HM) are increasingly used to prevent and treat various illnesses [1]. The prevalence of HM use in pregnancy has been reported in various countries, including Iran (67%) [2], Jordan (60%) [3], the United Kingdom (58%) [4], Iraq (54%) [5], Italy (48%) [6], Norway (40%) [7], Palestine (40%) [8], and Australia (34%) [9]. The popularity of HM among pregnant women is mainly because of the common belief that herbs are natural and free of any adverse effects that are usually associated with conventional drugs [10, 11]. HM can be obtained without prescription of a qualified healthcare practitioner in most countries, especially in the developing world where plants with medicinal properties can be easily purchased and herbs can also be obtained as unregulated food products, such as spices, that do not go through pharmaceutical regulatory processes [12]. Hence, there is a risk of adulteration with undeclared chemicals and contamination with heavy metals such as arsenic [13]. Additionally, herbs contain biologically active ingredients that have a potential to mediate pharmacological actions; thus, HM may produce adverse effects [14].

However, due to a lack of data on efficacy and safety of HM use in pregnancy, pregnant women have very little knowledge about the efficacy and safety of using these medicines [15]. Moreover, among the HM users in pregnancy, 39% of them used HM that were either potentially harmful to use in pregnancy or information on the safety was unavailable [16]. A recent multinational study, classifying safety of herbal medicines used in pregnancy, reported that out of 126 different HM, only 22% of these medicines were safe to use in pregnancy based on current scientific literature [17]. More recently, authors of this study have shown that out of 31 most commonly used modalities of HM by Asian pregnant women, 18 were potentially harmful to use in pregnancy [18]. Other researchers have tried to determine the possible effects of HM used in pregnancy on its outcome. Chuang et al. reported that consumption of certain Chinese HM in pregnancy was associated with increased incidence of congenital malformation of multiple organs [19]. Later, another report suggested that women using licorice and chamomile during pregnancy had higher incidence of threatening miscarriage and preterm labor, and newborns of herbal medicine users were smaller for their gestational age [20]. However, little is known about the possible effects of using HM during pregnancy on its outcome in low and middle income countries.

Moreover, in high income countries such as the USA, the UK and Australia, healthcare providers and consumers can report side effects of drugs, including HM, to the government authorities by filling out a prescribed form [21]. The process commonly known as Spontaneous Reporting in Pharmacovigilance. Whereas in the developing countries such as Bangladesh, there is no such mechanism neither in health facilities nor at community level. Consequently, the adverse effects experienced by consumers may remain undocumented. The aim of this research was to determine the prevalence of HM used in pregnancy by women in one region of Bangladesh, characteristics of its use, and possible adverse effects on the outcome of pregnancy.

## Methods

### Study setting and participants

This study was a descriptive cross-sectional in design, conducted at two public hospitals – Dhaka Mohanagar Hospital and Nazirabazar Maternity Hospital – in Dhaka, Bangladesh. Postpartum women before discharge from the hospitals were eligible to take part in the survey. Exclusion criteria were women who were in intensive care unit, in high dependency unit or were being managed for a severe condition. Women unable to speak or mentally unstable were also excluded. This study was part of a multinational research for which ethical approval was obtained from the Institutional Review Board on Human Subjects Research and Ethics Committees, Hanyang University, Seoul, Korea (HYI-17-067-2). Written informed consent was obtained from all participants.

### Survey instrument

After reviewing the literature [5, 20], the survey questionnaire was developed in English and then translated into Bengali language to suit the target population. The Bengali questionnaire was back translated into English to confirm the validity. Face validity of the questionnaire was done by two physicians in the target hospitals and a traditional medicine healthcare practitioner. The questionnaire was then pilot tested on a sample of 20 participants and based on the results, a few items were revised. Final version of the questionnaire comprised 30 items, with multiple choice, and open-ended questions (See Additional file 1: Survey questionnaire). It was divided into five sections. First section included general questions such as health status, mode of delivery, gravidity, exposure to secondary smoking, and antenatal care services. A question on maternal smoking was included in the questionnaire for pilot study, which showed no smoker out of 20 participants. Physicians in the target hospitals suggested to omit the question on the grounds that social norms and traditional values do not favor smoking by young girls or women in South Asian countries including Bangladesh [22]. Therefore, it was suggested that if such a question was included, there was a risk not to get any response or to get false positive or false negative answer. The question was, therefore, excluded, and question on secondary smoking was included. Multiple studies have found association between maternal exposure to passive smoking and low birth weight [23–25]. It is reported that pregnant women are consistently exposed to home smoking in many countries, especially in the developing countries where legislations on active and passive smoking are rather weak. High number of pregnant women exposed to home smoking is also reported from Asian countries including Bangladesh [26].

Second section comprised questions on morbidities during pregnancy (polyhydramnios, oligohydramnios, convulsions, fainting, high grade fever, severe anemia, premature rupture of water bag, severe headache, placenta previa, placental abruption gestational diabetes, hypertension, urinary tract infections, hyperemesis), complications during labor and maternal complications after childbirth (fetal distress, prolonged labor, abnormal presentation, profuse bleeding, urinary and fecal incontinence, severe anemia, smelly vaginal discharge, severe headache, high grade fever, convulsions, postpartum haemorrhage, postpartum depression, haemorrhoids). Third section included questions regarding herbal medicines use such as, modalities of herbal medicines and their indications, side effects if any, frequency of use, source of recommendation on herbal medicines use in pregnancy, disclosure of use with doctor or midwife, reason of use and non-use. To ensure that respondents only report HM used for medicinal purposes, the question was asked, “Did you use any herb or herbal preparation (syrup, paste, powder) for medicinal purposes or to improve your health during your last pregnancy?” Fourth section of the questionnaire included characteristics of the newborn baby such as birth weight, any birth defect and neonatal symptoms (neonatal jaundice, breathing problems, cardiac symptoms, tender or tense abdomen, convulsions, unconsciousness, bowel problems, lethargy or no activity). The fifth and final section asked questions related to sociodemographic characteristics of the participants. Monthly household income was categorized into three groups: low income (< $200), middle income ($201 - $400), and high income (> $400).

### Data collection

One supervisor (MAH) and four data collectors were trained and participated in the process of data collection. They were in regular contact with the main researcher (MA). Experienced female data collectors were recruited to make sure accurate data were collected. Experienced and female interviewers are more likely to be friendly and generate a good response rate [27]. A total of 300 women were invited to participate in the study when they were shifted to maternity wards after the childbirth. The participation was entirely voluntary and a complete anonymity was ensured. Information related to complications during labor, after birth, and newborn characteristics was collected with the help of hospital staff. Whereas, other questions were asked from the participants themselves. The survey was conducted from July 4 to August 12, 2017.

### Statistical analysis

The collected data were entered in the Microsoft Excel and were analyzed using software Statistical Package for Social Sciences (SPSS) v. 21. Respondents were categorized as users if they used at least one modality of herbal medicine in their previous pregnancy, whereas others were categorized as non-users. The descriptive statistics were computed as frequency and percentage. To determine the correlation for several characteristics between users and non-users of herbal medicines, Pearson’s Chi-square test and Fisher’s exact test were executed. *P*-value of less than 0.05 was considered statistically significant for all analyses.

## Results

### Sociodemographic characteristics

A total of 300 women were invited to the study and only 243 agreed to participate in the survey, generating a response rate of 81%. Data of 243 postpartum women were included in the final analysis. Sociodemographic characteristics of the study participants are presented in Table 1. Mean age of the respondents was 26.22 ± 5.54 years. Fifty-eight per cent aged 21 to 30 years, 88.1% belonged to urban areas, 63% had up to elementary school, 12.8% were employed, 65.4% were from middle income group, and 63.4% of the participants reported that it took less than 30 min to get to the nearest health facility.

**Table 1:** Sociodemographic characteristics of participants

Variables	Total = 243 *N* (%)	HM users =170 *N* (%)	Non-users =73 *N* (%)	*p*-Value
Mean age (years)	26.2 ± 5.5	26.2 ± 5.6	26.2 ± 5.5	
20 years and below	51 (21)	35 (20.6)	16 (21.9)	0.896
21–30 years	141 (58)	98 (57.6)	43 (58.9)	
31 years and above	51 (21)	37 (21.8)	14 (19.2)	
Residence				
Rural	29 (11.9)	17 (10)	12 (16.4)	0.156
Urban	214 (88.1)	153 (90)	61 (83.6)	
Education level				
Elementary school or under	153 (63)	113 (66.5)	40 (54.8)	0.084
High school or above	90 (37)	57 (33.5)	33 (45.2)	
Employed				
Yes	31 (12.8)	12 (7.1)	19 (26)	0.000 *X*^*2*^ = 16.510
No (housewife)	212 (87.2)	158 (92.9)	54 (74)	
Household income				
Low income	29 (12)	20 (11.8)	9 (12.3)	0.297
Middle income	159 (65.4)	116 (68.2)	43 (58.9)	
High income	55 (22.6)	34 (20)	21 (28.8)	
Time to the nearest health facility				
Less than 30 min	154 (63.4)	109 (64.1)	45 (61.6)	0.783
30 min to 1 h	76 (31.3)	53 (31.2)	23 (31.5)	
More than 1 h	13 (5.3)	8 (4.7)	5 (6.9)	

*HM*: Herbal Medicine

### Medical characteristics

Tables 2 and 3 presents medical characteristics of the study participants. Overall, 63.8% of the childbirths were conducted via normal delivery (includes forceps delivery), 77% of the childbirths were full term, 56.4% of women had one to 3 antenatal visits, 67.1% of the women were exposed to secondary smoking, 65.4% were multigravida, 45.3% women had experienced their first pregnancy between 20 to 24 years of age, 55.6% used HM prior to last pregnancy, and 40.7% experienced at least one morbidity during pregnancy.

**Table 2:** Medical characteristics of participants

Variables	Total = 243 *N* (%)	HM users =170 *N* (%)	Non-users =73 *N* (%)	*p*-Value
Prior use of Herbal Medicine				
Yes	135 (55.6)	102 (60)	33 (45.2)	0.033 *X*^*2*^ = 4.527
No	108 (44.4)	68 (40)	40 (54.8)	
Antenatal visits				
None	45 (18.5)	32 (18.8)	13 (17.8)	0.490
1 to 3 times	137 (56.4)	99 (58.2)	38 (52.1)	
4 or more times	61 (25.1)	39 (23)	22 (30.1)	
Exposed to passive smoking				
Yes	163 (67.1)	114 (67.1)	49 (67.1)	0.992
No	80 (32.9)	56 (32.9)	24 (32.9)	
Gravidity				
Primigravida	84 (34.6)	57 (33.5)	27 (37)	0.603
Multigravida	159 (65.4)	113 (66.5)	46 (63)	
Age at first pregnancy				
19 years or under	100 (41.1)	66 (38.8)	34 (46.6)	0.531
20 to 24 years	110 (45.3)	80 (47.1)	30 (41.1)	
25 years or above	33 (13.6)	24 (14.1)	9 (12.3)	
Morbidities during pregnancy				
Yes	99 (40.7)	75 (44.1)	24 (32.9)	0.102
No	144 (59.3)	95 (55.9)	49 (67.1)	

*HM*: Herbal Medicine

**Table 3:** Pregnancy outcomes of participants

Variables	Total = 243 *N* (%)	HM users =170 *N* (%)	Non-users =73 *N* (%)	*p*-Value
Perceived health status				
Good	172 (70.8)	124 (72.9)	48 (65.8)	0.259
Fair or worse	71 (29.2)	46 (27.1)	25 (34.2)	
Type of last delivery				
Normal (inc. forceps)	155 (63.8)	108 (63.5)	47 (64.4)	0.899
	88 (36.2)	62 (36.5)	26 (35.6)	
Pregnancy term				
Full term	187 (77)	133 (78.2)	54 (74)	0.469
Pre term	56 (23)	37 (21.8)	19 (26)	
Complications during and after childbirth				
Yes	96 (39.5)	70 (41.2)	26 (35.6)	0.416
No	147 (60.5)	100 (58.8)	47 (64.4)	
Birth weight				
Less than 2.5 kg	7 (2.9)	2 (2.3)	5 (6.8)	.067
2.5 to 3 kg	154 (63.4)	109 (64.1)	45 (61.6)	
More than 3 kg	82 (33.7)	59 (34.7)	23 (31.5)	
Neonatal symptoms				
Yes	128 (52.7)	95 (55.9)	33 (45.2)	.126
No	115 (47.3)	75 (44.1)	40 (54.8)	

*HM*: Herbal Medicine

No significant difference between users and non-users of HM were found in terms of morbidities during pregnancy, complications around childbirth, except for convulsions during pregnancy (add % and p value) and neonatal symptoms (add % and *p* value)

### Characteristics of neonates

Characteristics of neonates are presented in Table 3. In terms of birth weight, 63.4% were between 2.5 to 3 kg, followed by more than 3 kg (33.7%). Neonatal symptoms were reported in 52.7% of the cases. There were seven (2.9%) cases of birth problems that included one case of cardiac anomaly and six cases of congenital nasolacrimal duct obstruction (not shown in tables). Out of 243 childbirths, 50.2% were baby boys and 49.8% were baby girls (not shown in tables).

### Characteristics of herbal medicine users

Out of 243 respondents (Tables 1 and 2), 70% used at least one modality of HM in their last pregnancy. HM were more commonly used among women who aged 21 to 30 years (57.6%), lived in urban areas (90%), had completed education up to elementary school (65.9%), unemployed (92.9%), belonged to middle income group (68.2%), and could access the nearest health facility in less than 30 min (64.1%). Chi square analysis showed that use of HM during pregnancy was significantly associated with women who were unemployed (*X*^*2*^ = 16.510; *p* value = 0.000).

Regarding medical characteristics, 60% of HM users had used HM prior to last pregnancy, had one to three antenatal care visits (58.2%), were exposed to passive smoking at home (67.1%), were multigravida (66.5%), and experienced first pregnancy between 20 to 24 years (47.1%). Chi square analysis showed that prior use of HM (*X*^*2*^ = 4.527; p value = 0.033) was significantly associated with its use during pregnancy.

### Use of herbal medicines and pregnancy outcome

Use of HM in terms of pregnancy outcome is presented in Table 3. No significant association was found between use of HM and pregnancy outcome.

### Modalities of herbal medicines and self-reported indications

Self-reported indications according to each modality of HM are presented in Table 4. The most frequently used HM – ginger – was used for nausea/vomiting (76.5%), cold/flu (30.4%), and cough (2.9%). Black seed was used for nausea/vomiting (60.3%), as galactagogue (39.7%), and for allergies (3.2%). Lemon tea was used for cough (59.2%) and nausea/vomiting (46.9%).

**Table 4:** Reported indications of herbal medicine according to each modality (multiple choice)

Herbal modality	Reported indication	*N* (%)
Ginger - *Zingiber officinale* (*N* = 102)	Nausea/vomiting	78 (76.5)
	Cold/flu	31 (30.4)
	Cough	3 (2.9)
Black seed - *Nigella sativa* (*N* = 63)	Nausea/vomiting	38 (60.3)
	Galactagogue	25 (39.7)
	Allergies	2 (3.2)
Lemon tea (*N* = 49)	Cough	29 (59.2)
	Nausea/vomiting	23 (46.9)
Prune - *Prunus domestica* (*N* = 29)	Nausea/vomiting	23 (79.3)
	Hypertension	4 (13.8)
	Constipation	2 (6.9)
Mustard oil - *Brassica nigra L.* (*N* = 29)	Cold/flu	18 (62.1)
	Improve immunity	7 (24.1)
	Constipation	4 (13.8)
Garlic - *Allium sativum* (*N* = 26)	Hypertension	12 (46.2)
	Cold/flu	12 (46.2)
	Abdominal pain	3 (13)
Betel nuts - *Areca catechu* (*N* = 23)	As relaxant	20 (87)
	Nausea/vomiting	3 (13)
	Heartburn	1 (4.3)
Peppermint - *Mentha piperita* (*N* = 15)	Abdominal pain	8 (53.3)
	Heartburn	4 (26.7)
	Headache	2 (13.3)
	Diabetes mellitus	1 (6.7)
Myrobalan - *Terminalia chebula* (*N* = 11)	Heartburn	5 (45.4)
	Constipation	5 (45.4)
	Nausea	2 (18.2)
Turmeric - *Curcuma longa* (*N* = 11)	Skin condition	8 (72.7)
	Anti-inflammatory	3 (27.3)
*Aloe vera* (N = 4)	Stomach upset	3 (75)
	Constipation	1 (25)
Olive oil (*N* = 3)	Massage	3 (100)
Neem - *Azadirachta indica* (*N* = 3)	Skin disease	3 (100)
Bitter gourd - *Momordica charantia* (*N* = 2)	Diabetes mellitus	2 (100)
Syrup Alkuli [*Cichorium endivia, Tribulus terrestris, Foeniculum vulgare, Cucumis melo*] (*N* = 2)	Urinary tract infection	2 (100)

### Self-reported side effects

The most frequently reported side effects after its use during pregnancy were dry mouth (6), nausea/vomiting (3), drowsiness (3), diarrhea (2), and palpitation (1).

### Recommendations of herbal medicines

As presented in Table 5, the main recommending source for HM is the members of their family, friends or neighbor (71.1%), followed by herbalist (28.2%), Islamic/religious text (24.7%), and midwife/health worker (20%).

**Table 5:** Patterns of herbal medicine use during pregnancy

Variables	Number (%)
Recommending source (*N* = 170) – multiple choice	
Family/friends/neighbor	121 (71.1)
Herbalist	48 (28.2)
Islamic/religious text	42 (24.7)
Midwife/health worker	34 (20.0)
Newspaper/magazine	16 (9.4)
TV/radio/internet	10 (5.9)
Doctor	9 (5.3)
Frequency of use (N = 170)	
Occasionally	92 (54.1)
Daily	64 (37.6)
Weekly	7 (4.1)
Twice or more a week	5 (2.9)
Only once	2 (1.2)
Disclosed with doctor or midwife (N = 170)	
Yes	43 (25.3)
No	127 (74.7)
Reason of non-disclosure (*N* = 125)	
Should have informed but forgot	65 (52.0)
Doctor did not ask	39 (31.2)
Afraid of doctor’s response	12 (9.6)
It was not important	9 (7.2)
Reason for using herbal medicines (*N* = 169) – multiple choice	
It is cheap and accessible	159 (94.1)
I believe it is safe	116 (68.6)
I believe it is effective	22 (13.0)
Family tradition/culture	10 (5.9)
Unsatisfied with modern medicine	4 (2.4)
Reason for not using herbal medicines (*N* = 73) – multiple choice	
My family did not let me use	45 (64.1)
My doctor/midwife did not let me use	39 (53.4)
It is not effective	15 (20.5)
I am satisfied with modern medicine	10 (13.7)

### Frequency of herbal medicines use

Among 170 users of herbal medicine (Table 5), 54.1% reported using it occasionally, daily (37.6%), weekly (4.1%), twice or more a week (2.9%), and only once (1.2%).

### Disclosure of using herbal medicine

Among 170 users (Table 5), only 25.3% disclosed it to their doctor or midwife. The most frequently reported reasons for non-disclosure were forgot to inform (52%), followed by doctor did not ask (31.2%), afraid of doctor’s response (9.6%), and it was not important to disclose (7.2%).

### Reasons for using and not using herbal medicines

Reasons for using HM during pregnancy are presented in Table 5. Among 169 users, the most frequently reported reasons for using HM were it was cheap and accessible (94.1%), followed by belief that it was safe (68.6%), belief that it was effective (13%), family tradition/culture (5.9%), and unsatisfied with modern medicine (2.4%). Among 73 non-users, the most frequently reported reasons for not using HM during pregnancy were family did not let them use (61.6%), followed by doctor or midwife did not let them use (53.4%), belief that it was not effective (20.5%), and satisfied with modern medicine (13.7%).

## Discussion

This is the first study to investigate use of HM among Bangladeshi women and its possible effects on the outcome of pregnancy. The study followed cross-sectional design and data were collected via survey on postpartum women in two maternity hospitals in Dhaka, Bangladesh. The results show that the use of HM in pregnancy is common in Bangladesh.

It was found that 70% of the women used at least one herbal product during their last pregnancy. This prevalence rate is significantly quite high considering the fact that more than 88% of the sample resided in urban areas, and majority of the participants lived near to a health facility. These results suggest higher prevalence than previously reported from Asian and non-Asian countries [8, 20, 28–31]. The difference in the prevalence of HM use could be explained by several factors such as culture, socio-demographic factors, and use of health care services.

Likely predictors of using HM among Bangladeshi women in pregnancy were unemployment and use of HM prior to last pregnancy, which is consistent with previous studies [5, 32]. The most common reasons to use HM were that these were cheap, accessible, and safe. A study from Ethiopia also reported that most of the women used HM in pregnancy because they considered these cheap and accessible [33]. Elsewhere, it has been reported that HM are consumed during pregnancy because these are considered natural and safe [34]. Moreover, the findings suggest that most of the women used HM occasionally or daily throughout their previous pregnancy, consistent with previous studies [3, 5]. These findings imply that women used HM occasionally when they needed to manage their symptoms or used on daily basis to stay healthy.

In this study, family, friends, and neighbors were the main source of recommendation for use of herbal medicines in pregnancy. Similar findings were reported by studies conducted in the United Kingdom and Iraq [5, 35]. These results are conceivable as family and friends are easily accessible, and usually the most trusted members of a society. Furthermore, a majority of study participants did not inform healthcare providers about their use of HM in pregnancy. When asked for the reason of this non-disclosure, most of them responded that they forgot and the healthcare providers did not ask them, consistent with a previous report [5]. It shows respondents’ lack of awareness about safety issues regarding use of HM in pregnancy. The findings also reflect poor communication between healthcare providers and their patients.

Ginger was the most frequently used modality of HM, and it was primarily used for relief of nausea/vomiting and cold/flu. Ginger has been widely used to treat pregnancy related morning sickness. However, there is a need for more data because its clinical value and safety profile in treating nausea and vomiting in early pregnancy is still unknown [36]. Black seed was frequently used for nausea/vomiting and as a galactagogue. It is suggested that this herb’s potential to increase the milk flow could be due to its constituents: lipid portion and hormonal structures [37]. Lemon tea was primarily used to soothe cough during pregnancy. Lemon may be suitable for treating mild cough during pregnancy [38]. However, it should be ensured that there is no underlying cause such as asthma or bacterial infection. Apart from garlic, a few women consumed prunes to treat their hypertension. A placebo controlled clinical study from Pakistan reported significant reduction in blood pressure following use of prunes [39].

Although modalities of HM reported in this study may have some benefit, certain herbs could be potentially harmful to use in pregnancy or the information about their safety may be lacking. For instance, information on safe use of black seed, prunes, and mustard oil during pregnancy is lacking and maternal use of betel nuts, terminalia, and turmeric is not recommended at higher doses. Indeed, a recent study reported that betel nuts chewing during pregnancy was associated with increased chances of anemia. [40]. Turmeric and terminalia, if ingested in doses higher than commonly found in food, can stimulate uterus and may trigger its emmenagogic and abortifacient effects during pregnancy [41–43].

Participants of this study were asked to report any side effect following their use of HM in pregnancy. The findings show that among 170 users, side effects were reported in 15 instances (8.8%). In previous studies, the reported side effects ranged between 3.7 and 18% among HM users [20, 44]. Few participants of this study reported dry mouth and nausea/vomiting following their consumption of garlic in pregnancy. Similar adverse effects have been reported in the literature [45]. Moreover, two cases of diarrhea were reported following consumption of myrobalan or *Terminalia chebula* which is used to alleviate constipation [46]. In this study, five respondents used it for this symptom. It is possible that high doses of terminalia were taken to relieve severe constipation, resulting in diarrhea.

This study did not find any significant difference between users and non-users of HM in terms of morbidities during pregnancy, complications around childbirth, and neonatal symptoms. To evaluate the data even further, deep analyses of each herbal medicine were conducted to see their relationship with maternal and neonatal characteristics. No significant adverse effect of using individual modality of HM was found on the outcome of pregnancy. The number of cases with complications during childbirth was too small to make reliable inference based on statistical analysis. Further research will need to clarify if there is a relationship, using a larger sample size. Furthermore, there were six cases of blocked nasolacrimal duct which is a common neonatal condition.

This study showed that women during pregnancy used HM a lot. Information given by physician and midwives could help the pregnant women to use the herbs that have less risk [18]. Therefore, healthcare practitioners should maintain proper communication with their patients to ensure their health and safety [47]. Moreover, physician and midwives should not forget to ask pregnant women on HM use, and write it in the medical records, as it is written for drugs. Reports of side effects and recommendations by unqualified people suggest that there is need to establish an integrative pharmacovigilance of HM within communities and health facilities, especially in the maternity clinics. So that data regarding use of HM and herbs, their indications, and potential effects can be gathered. Such data would contribute to evidence based policymaking and health awareness campaigns in maternal and child health programs.

This study has several limitations. The data was collected from two maternity hospitals of Dhaka city. Therefore, it does not represent the entire population of Bangladesh and the findings cannot be extrapolated to whole country. Moreover, the study could not estimate the dose and frequency of ingestion of individual HM. There could be a recall bias in the questionnaire as it was retrospective data. However, asking before discharge could decrease this risk, as it was very close to the pregnancy. Another limitation is that the whole data is self-reported, even if there was the help of medical staff. A separate questionnaire should have been distributed to physicians and midwifes in order to be sure that it is not only self-reported. The major strength of this study is that this is the first attempt to see the relationship between maternal use of HM and outcome of pregnancy in South Asian region.

## Conclusion

The results from this first study in Bangladesh highlight that the use of HM during pregnancy is common, and frequently recommended by informal caregivers. The high prevalence of HM use among pregnant women without consultation of a qualified healthcare practitioner highlights that physicians and midwives should ask about HM use. Lack of ample scientific evidence to support safety and efficacy of HM use in pregnancy demands health programs to focus on creating awareness among healthcare practitioners and community people on safe use of HM. Moreover, as previous use of HM before pregnancy is associated with use during pregnancy, general practitioners could already give some advices to women before pregnancy. High prevalence of HM use among pregnant women in our study warrants further studies in other countries.

## Additional file

Additional file 1: Survey questionnaire. (DOCX 30 kb)

## Abbreviations

HM: Herbal Medicine
