# Supplementary material for: Herbal medicine use by pregnant women in Bangladesh: a cross-sectional study
Source: BMC Complement Altern Med. 2018 Dec 13;18:333. doi: 10.1186/s12906-018-2399-y (PMC6293557; doi:10.1186/s12906-018-2399-y)
Supplement: Supplementary file 1 — Survey questionnaire. (DOCX 30 kb) [file 12906_2018_2399_MOESM1_ESM.docx]

**Section A: Questions on your medical characteristics:**

1. In general, would you say your current health is:

| □①Excellent | □②Good | □③Fair | □④Poor | □⑤Very poor |
| --- | --- | --- | --- | --- |

2. Type of delivery of last pregnancy

| □①Normal | □②Caesarian | □③ Forceps | □④Vacuum | □⑤ Other ___________ |
| --- | --- | --- | --- | --- |

3. Obstetric history (write appropriate numbers in each box)

| 3-1. Number of times you have become pregnant | 3-2. Age at first pregnancy |
| --- | --- |
|  |  |
|  |  |

4. Does anyone smoke cigarette or shisha/hookah in your home?

□① Yes □② No

5. Number of times antenatal care services received during last pregnancy

□① None □② 1 to 3 times □③ 4 or more times

6. Check (√) the complications, if you experienced/diagnosed, during last pregnancy (may select more than one) [See the pregnancy file if possible]

| □ 1) Preeclampsia/hypertension | □ 2) Oligohydramnios |
| --- | --- |
| □ 3) Polyhydramnios | □ 4) Convulsions |
| □ 5) Fainting | □ 6) High grade fever |
| □ 7) Urinary tract infection | □ 8) Gestational diabetes |
| □ 9) Severe anemia | □ 10) Premature rupture of water bag |
| □ 11) Severe headache | □ 12) Hyperemesis gravidarum |
| □ 13) Placenta previa | □ 14) Placental abruption |
| □ 15) Others ______________________ | □ 16) None |

7. Check (√) the complications, if you experienced/diagnosed, during labor and/or after your last delivery (may select more than one) [See the pregnancy file if possible]

| □ 1) Prolonged labor | □ 2) Abnormal presentation |
| --- | --- |
| □ 3) Fetal stress | □ 4) Profuse bleeding |
| □ 5) Blood transfusion | □ 6) Urinary or fecal incontinence |
| □ 7) Hypertension | □ 8) Hemorrhoids |
| □ 9) Severe anemia | □ 10) Smelly vaginal discharge |
| □ 11) Severe headache | □ 12) High grade fever |
| □ 13) Convulsions | □ 14) Postpartum depression |
| □ 15) Others ______________________ | □ 16) None |

**Section B: Use of Herbal Medicine during your most recent pregnancy**

Explanation: Herbal Medicine means ‘Any herb (such as ginger, black seed, lemon tea) or herbal preparation (syrup, paste, powder) used during your most recent pregnancy either self-prescribed by you or recommended by family/friends/other person or health professional in order to manage any symptoms, prepare for delivery or to support baby’s development’.

8. Did you ever use any herb (such as ginger, black seed, lemon tea) or herbal preparation (syrup, paste, powder) to improve your health before your last pregnancy?

□ ① Yes □ ② No

9. Did you use any herb (such as ginger, black seed, lemon tea) or herbal preparation (syrup, paste, powder) to improve your health during your last pregnancy?

□ ① Yes □ ② No (go to Question 18)

10. Check (√) all of the following herbs that you used, for which symptoms, to manage your health, to prepare for labor and to support baby’s development. (may select more than one)

| Herbs | Indications (reason for use) | | | |
| --- | --- | --- | --- | --- |
| 1) Ginger (or its tea) | □Cough | □Cold/flu | □Heartburn | □Vomiting/nausea |
| 2) Black seed | □Asthma/breathing problems | □Vomiting/nausea | □Allergies | □Other |
| 3) Honey (or its tea) | □Cough | □Vomiting/nausea | □Facilitate delivery | □Other________ |
| 4) Lemon tea | □Cough | □Vomiting/ nausea | □Heartburn | □Other________ |
| 5) Myrobalan | □Asthma/breathing problems | □Heartburn | □Constipation | □Other________ |
| 6) Prune | □Hypertension | □Constipation | □Vomiting/ nausea | □Other________ |
| 7) Betel nuts | □Vomiting/ nausea | □Heartburn | □As relaxant | □Other________ |
| 8) Mustard oil | □Cold/flu | □Constipation | □Improve immunity | □Other________ |
| 9) Garlic (lahsun) | □Abd. pain | □ Cold/flu | □Fatigue | □Hypertension |
| 10) Turmeric (haldi) | □Cough | □Cold/flu | □Skin condition | □Anti-inflammatory |
| 11) Peppermint (pudeena or its tea) | □Abd. pain | □Cold/flu | □Heartburn | □Other________ |
| 12) Olive oil | □Skin condition | □Massage | □Nutrition | □Other________ |
| 13) Aloe vera | □Skin/hair | □Constipation | □Stomach upset | □Other________ |
| 14) Other herbs  ___________________ | □ _____________ | □ _____________ | □ _____________ | □ _____________ |

11. How frequently did you use the above herbal medicine during pregnancy?

| □① Daily | □②two or more times a week | □③ Weekly |
| --- | --- | --- |
| □④ Occasionally | □⑤ Only once |  |

12. Did you experience any of the following effects after use of the above herb or herbal preparation during last pregnancy? (may select more than one)

| □ 1) Abdominal pain | □ 2) Diarrhea |
| --- | --- |
| □ 3) Constipation | □ 4) Nausea/vomiting |
| □ 5) Dry mouth | □ 6) Urine problems |
| □ 7) Skin rash | □ 8) Severe headache |
| □ 9) Fatigue | □ 10) Dizziness/drowsiness |
| □ 11) Sedation/sleepiness | □ 12) Palpitation/hypertension |
| □ 13) Edema | □ 14) Allergic reaction |
| □ 15) Loss of appetite | □ 16) Other__________________ |
| □ 17) None (go to Question 15) |  |

13-1. Name of herb or herbal preparation that caused above effects: _____________________

14. Who recommended herbal medicine you used during pregnancy (may select more than one)

| □ ① Family/friends/neighbor | □ ② Herbalist | □ ③ Doctor |
| --- | --- | --- |
| □ ④ Midwife or health worker | □ ⑤ Newspaper/magazine | □ ⑥ TV/radio/internet |
| □ ⑦ Islamic/religious text | □ ⑧Other_____________________ | |

15. Did you inform your doctor or midwife about herbal medicine you used during pregnancy?

□ ① No □ ② Yes (go to Question 18)

16. If no, why you didn’t inform your doctor or midwife?

| □ ① Doctor didn’t ask | □ ② It was not important |
| --- | --- |
| □ ③ Afraid of doctor’s response | □ ④ Should have informed but I forgot |
| □ ⑤ Other____________________________________ | |

17. What is the reason you used above herbal medicine during pregnancy? (may select more than one)

| □①I believe it’s effective | □②I believe it’s safe | □③Family, tradition or culture |
| --- | --- | --- |
| □④ It’s cheap and accessible | □⑤I am not satisfied with modern medicine | |

18. What is the reason you did not use above herbal medicine during pregnancy? (may select more than one)

| □①It’s not effective | □②It’s not safe | □③I am satisfied with modern medicine |
| --- | --- | --- |
| □④It’s expensive and difficult to get | □⑤My family didn’t let me use | □⑥My doctor/nurse didn’t let me use |

**Section C: Characteristics of the Newborn baby [see the newborn card if possible]**

19. Gestational age at birth (please write exact number in weeks) __________ weeks

20. Gender of the newborn

□ ① Boy □ ② Girl

21. Weight of the newborn (please write exact number in grams) ________________ grams

22. Any congenital malformations/birth defects to the newborn? (may select more than one) [see the newborn card if possible]

| □ 1) Nervous system anomalies (anencephaly, hydrocephalus etc.) | |
| --- | --- |
| □ 2) Cleft lip/palate | □ 3) Cardiac anomalies |
| □ 4) Genital organ anomalies | □ 5) Down Syndrome |
| □ 6) Tongue/mouth/pharynx | □ 7) Imperforate anus |
| □ 8) Congenital malformations of face/neck | □ 9) Other____________________ |
| □ 10) None | |

23. Check the symptoms if the newborn baby experienced (may select more than one) [see the newborn card if possible]

| □ 1) Breathing problems | □ 2) Appearance (blue or pale) |
| --- | --- |
| □ 3) Newborn jaundice (yellow skin, eyes) | □ 4) Convulsions/unconsciousness |
| □ 5) Excessively irritable and crying | □ 6) Pus, bleeding around umbilicus |
| □ 7) Tender or tense abdomen | □ 8) Fever |
| □ 9) Body gets cold easily | □ 10) Any infections |
| □ 11) Bowel problems (blood in bowel, diarrhea) | |
| □ 12) Activity (weak suck on feeding, lethargic, no muscle activity) | |
| □ 13) Cardiac problems (abnormal heart rate or blood pressure) | |
| □ 14) Others _____________________________________ | □ 15) None |

**Section D: Sociodemographic characteristics of the study participant**

1. What is your age? completed years ___________
2. Where do you live?

| □ ① Rural area | □ ② Urban area |  |
| --- | --- | --- |

26. What is the highest level of education you have completed?

| □ ①No education (Illiterate) | □ ②Elementary | □ ③High school | □ ④College/university |
| --- | --- | --- | --- |

1. Are you employed?

| □ ①Yes | □ ②No (housewife) |  |
| --- | --- | --- |

28. What is monthly income in your home?

| □ ①Less than TK 8,000 | □ ②TK 8,001 to 15,000 | □ ③TK 15,001 to 25,000 |
| --- | --- | --- |
| □ ④TK 25,001 to 35,000 | □ ⑤TK 35,001 and above |  |

29. Time to get to the nearest health facility

| □ ①Less than 30 minutes | □ ②30 minutes to 1 hour | □ ③More than 1 hour |
| --- | --- | --- |

---------------------------------------------THANK YOU-----------------------------------------
